# Generative AI usage and employee innovation performance: the chain mediation mechanism of resource acquisition, integration, and activation

**DOI:** 10.3389/fpsyg.2026.1923950

**Published:** 2026-09-07

**Authors:** Da Teng, Yaoyao Shen, Ruixue Yuan, Weiyue Wang

**Affiliations:** 1 College of Economics and Management, Beijing University of Chemical Technology, Beijing, China; 2 Birmingham Business School, University of Birmingham, Birmingham, United Kingdom

**Keywords:** cognitive appraisal theory, conservation of resources theory, employee innovation performance, generative artificial intelligence, serial mediation, work stress

## Abstract

**Background:**

Generative artificial intelligence is profoundly reshaping knowledge work, yet the cognitive mechanisms through which AI use relates to employee innovation performance remain underexplored.

**Objective:**

Integrating Conservation of Resources theory and Cognitive Appraisal Theory, this study proposes that generative AI use is associated with innovation performance through a three-stage partially serial mediation pathway: resource acquisition (cognitive divergence), resource integration (cognitive elaboration), and resource activation (challenge appraisal), with work stress moderating each stage.

**Methods:**

Survey data from 612 knowledge workers were analyzed using structural equation modeling and Monte Carlo bootstrap methods.

**Results:**

(1) Generative AI use is positively associated with employee innovation performance; (2) the three-stage serial mediation model received support, with indirect effects accounting for 72.9% of the total effect; and (3) work stress moderation exhibits a stage-dependent pattern: non-significant at the resource acquisition stage but significantly strengthening mediation efficiency at the integration and activation stages.

**Conclusion:**

This study advances a chain mediation framework that offers a more coherent theoretical account of AI-enabled cognitive transformation and provides organizations with specific intervention points for shifting from tool adoption to cognitive empowerment.

## 1 Introduction

Generative AI is already changing knowledge work. Large language models can pull up information, draft solutions, and offer multiple perspectives in seconds (Brynjolfsson et al., 2025). But here is the practical question: how does using these tools actually lead to better innovation performance for employees? And when does this link get stronger or weaker? Recent experiments show that AI can boost both output quantity and quality (Noy and Zhang, 2023). Simply giving employees access to AI, however, is not the same as seeing them innovate with it. We still do not know much about the cognitive pathways involved. We also do not know which situational conditions determine whether AI use turns into real innovative behavior.

Researchers have increasingly examined how AI shapes employee innovation, yet this literature has developed along largely parallel tracks defined by the roles AI is understood to play. One prominent line treats AI primarily as an information provider and knowledge retriever—tools that expand employees’ information search space and increase the volume and quality of task output (Noy and Zhang, 2023; Brynjolfsson et al., 2025). A second line positions AI as a creative partner and solution generator, showing that AI participation reshapes idea generation patterns and collaborative problem-solving dynamics (Boussioux et al., 2024; Sun et al., 2025; Zhao et al., 2025). A third line conceptualizes AI as a process facilitator that restructures daily work routines, task flows, and role boundaries (Chu et al., 2025; Mariani and Dwivedi, 2024). More recently, a fourth perspective has emerged that recognizes AI as a double-edged resource, simultaneously supplying valuable capabilities and triggering threat perceptions such as skill atrophy and job insecurity (Guo et al., 2025; Bankins et al., 2024; Dell'Acqua et al., 2026).

Across these role-based perspectives, a shared blind spot persists. Existing research treats the cognitive pathway from AI use to innovation performance as either a direct pipeline or a single-step cognitive transfer. Without specifying the intermediate stages through which raw AI outputs are progressively transformed into innovative behavior, interventions designed to foster AI-assisted innovation remain blunt—investors and managers know that AI matters but lack a roadmap for how employees actually convert AI capabilities into creative action. We refer to this as the transformation problem. Existing models largely treat the AI-innovation link as either a direct pipeline or a single cognitive jump, leaving the sequential logic that connects resource acquisition, knowledge integration, and behavioral activation unexplained. Theoretical perspectives also remain relatively separate. Most studies adopt a single theoretical lens (Mariani and Dwivedi, 2024), each illuminating one facet of the process rather than resource accumulation and cognitive transformation together. Boundary conditions have not received adequate attention either. Work stress is a situational factor that nearly all knowledge workers experience, yet no study has systematically tested whether it moderates different stages of the mediation process in different ways.

To fill these gaps, we bring together Conservation of Resources theory (COR; Hobfoll, 1989, 2001; Hobfoll et al., 2018) and Cognitive Appraisal Theory (Lazarus and Folkman, 1984). Our framework says that generative AI use improves employee innovation performance through a three-stage serial mediation chain. The stages are resource acquisition (cognitive divergence), resource integration (cognitive elaboration), and resource activation (challenge appraisal). Work stress moderates each stage. We collected survey data from 612 knowledge workers. We then confirmed the serial mediation chain with structural equation modeling and Monte Carlo bootstrap. The results show that internal cognitive mechanisms carry most of the total effect. Work stress moderates the chain, but its effect depends on the stage.

Our study matters for both theory and practice. On the theory side, the three-stage serial mediation model addresses the transformation problem. It tracks how cognitive resources that AI provides become converted into actual innovative behavior. Existing models have examined parts of this conversion, but none have traced the full chain. By placing COR theory and Cognitive Appraisal Theory at different points along the same chain, we also suggest a workable strategy for combining resource and cognitive perspectives in technology research. The stage-specific moderation pattern adds nuance too. It shows that the same situational variable can have different effects at different cognitive stages. On the practice side, organizations that roll out AI should think less about pushing tools and more about empowering resources. They should also manage stress differently depending on which cognitive stage employees are in, if they want to see real innovative output.

## 2 Theoretical background and research hypotheses

### 2.1 Theoretical background

#### 2.1.1 Prior research on human-AI collaboration and innovation

Over the past decade, scholarly interest in how artificial intelligence reshapes knowledge work has grown rapidly. Early research focused primarily on productivity metrics, documenting efficiency gains and output improvements associated with AI adoption. More recent work has shifted toward understanding the psychological and organizational mechanisms that mediate AI’s effects on employee outcomes, particularly innovation. Despite this expansion, the literature remains fragmented across disciplines and theoretical traditions, making it difficult to identify shared assumptions and collective blind spots. To locate the present contribution precisely, this subsection systematically reviews prior research on human-AI collaboration and innovation along three organizing dimensions: the roles AI assumes in collaborative settings, the forms of human-AI interaction through which these roles are enacted, and the effects of AI on innovation-related outcomes.

Prior research can be organized around four conceptualizations of AI’s role in the innovation process. The first and most prevalent treats AI as an information provider and knowledge retriever. Noy and Zhang (2023) showed that AI-assisted writing reduces task completion time and raises output quality in professional tasks, while Brynjolfsson et al. (2025) reported efficiency gains in customer service work. These studies document efficiency and productivity outcomes such as output volume, speed, and technical accuracy. The second positions AI as a creative partner and solution generator. Boussioux et al. (2024) found that human-AI co-creation produces more novel and diverse candidate solutions than human-only approaches, and Sun et al. (2025) demonstrated that generative AI use enhances divergent thinking and idea novelty in field settings. This stream chiefly tracks creativity outcomes including idea diversity and exploratory search. The third frames AI as a process facilitator and task restructuring agent. Chu et al. (2025) examined how AI restructures artistic innovation routines, and Mariani and Dwivedi (2024) discussed how AI adoption changes daily work flows and task allocation. Research in this domain focuses on workflow and implementation outcomes such as task restructuring and the reallocation of cognitive effort. The fourth and more recent conceptualization recognizes AI as a double-edged resource. Guo et al. (2025) noted the mixed blessings of AI-driven resources, documenting both productivity gains and technology anxiety, while Dell'Acqua et al. (2026) observed jagged frontier effects in which AI helps below-average performers but may complicate tasks for top performers. Bankins et al. (2024) further noted that AI can trigger perceived skill obsolescence and ambivalent engagement. This emerging line documents ambivalent outcomes in which productivity gains co-occur with psychological threat.

These AI roles are enacted through distinct interaction modalities observed in prior research. One pattern describes instrumental deployment in which employees invoke AI for specific subtasks while retaining oversight of its outputs (Venkatesh et al., 2003; Noy and Zhang, 2023). In this mode, the technology remains subordinate to user intent, functioning primarily as a targeted instrument for information retrieval or solution generation. A second pattern involves reciprocal collaboration in which employees and generative AI interact as work partners. Li et al. (2025), for example, found that this collaborative relationship positively affects employees’ work engagement and family outcomes, suggesting that AI functions not merely as a tool but as an active contributor to shared goals. A third pattern entails deep cognitive integration in which AI suggests alternatives or offers perspectives that expand the user’s mental model, augmenting human cognitive capabilities rather than replacing them (Dell'Acqua et al., 2026). These interaction forms are not mutually exclusive, and an employee may shift among them within a single task. However, each form implies a different locus of cognitive agency and, consequently, a different pathway through which AI outputs become incorporated into innovative behavior.

Across the four roles and three interaction patterns reviewed above, AI’s effects on innovation-related outcomes cluster into three broad domains. Efficiency-oriented research, represented by Noy and Zhang (2023) and Brynjolfsson et al. (2025), documents gains in output volume, speed, and technical quality. Creativity-oriented research, represented by Boussioux et al. (2024) and Sun et al. (2025), tracks improvements in idea novelty, diversity, and exploratory search. Implementation-oriented research, represented by Chu et al. (2025) and Mariani and Dwivedi (2024), examines workflow restructuring, task automation, and the redirection of cognitive effort toward higher-order work. A smaller but growing body, represented by Guo et al. (2025), Bankins et al. (2024), and Dell'Acqua et al. (2026), records ambivalent outcomes in which productivity gains co-occur with technology anxiety and perceived skill obsolescence.

While these role-based, interaction-based, and outcome-based insights have expanded the literature, no study has modeled the cognitive pathway from AI use to innovation as a sequential multi-stage process. The following sections introduce the theoretical framework designed to trace this pathway (Table 1).

**Table 1:** AI roles in innovation.

AI role	Interaction modality	Key findings	Research gap
Information provider and knowledge retriever	Tool-based invocation; user retains full control	• Reduces task completion time and raises output quality • Efficiency and productivity gains (output volume, speed, accuracy)	Does not model the cognitive transformation process from information access to innovative behavior
Creative partner and solution generator	Collaborative reciprocity; human and AI alternate contributions	• Generates more novel and diverse candidate solutions • Enhances divergent thinking (idea novelty, diversity)	Does not explain how generated ideas are cognitively integrated and activated into implementation
Process facilitator and task restructuring agent	Augmentative integration; AI expands the user’s mental model without replacing human agency	• Restructures innovation routines and workflows • Redistributes tasks and redirects cognitive effort toward higher-order work	Does not trace how workflow changes translate into stage-dependent cognitive appraisals
Double-edged resource	Shifts among modalities depending on task context	• Productivity gains coexist with technology anxiety • Triggers perceived skill obsolescence (ambivalent outcomes)	Does not explain when and why AI use is appraised as challenge versus hindrance

#### 2.1.2 Conservation of resources theory

Conservation of Resources theory (COR) explains how people gain, protect, and spend their personal resources (Hobfoll, 1989). The theory builds on two basic ideas. First, losing a resource hurts more than gaining the same resource helps. This is the primacy of loss principle. Second, people must invest resources to gain new ones or to prevent further losses. This is the resource investment principle. Hobfoll (2001) later added the idea of resource gain spirals. Once someone gains an initial resource, it becomes easier to accumulate more resources, which creates a self-reinforcing upward cycle. Organizational researchers have already used COR theory to explain work engagement, innovative behavior, and how employees cope with stress (Hobfoll et al., 2018; Halbesleben et al., 2014). But can this theory also explain what happens when employees use generative AI? The resources at stake here are different from traditional workplace resources such as supervisor support or team climate, so the extension is not automatic. We think COR theory fits the generative AI context for three reasons. First, COR theory’s resource categories (object resources, condition resources, personal resources, and energy resources; Hobfoll, 1989, 2001) include the cognitive resources that AI provides. Generative AI delivers what we would call functional cognitive resources: cross-domain information, alternative solutions, and novel perspectives that directly add to what employees already know. These resources fall within COR theory’s personal resource category. Second, the step-by-step shift from resource acquisition to resource integration and activation mirrors COR theory’s resource gain spiral (Hobfoll, 2001). The spiral logic is that early gains set the stage for later gains. In our model, the cognitive resources that AI supplies create the basis for deeper knowledge integration, which in turn enables people to act. Third, COR theory has already been used to explain employee innovation behavior in organizations. Halbesleben et al. (2014) showed that resource investment and gain spirals predict innovative work behavior, and Hobfoll et al. (2018) explicitly called for extending COR theory to technology-empowered workplaces. Applying COR theory to generative AI answers that call directly. In short, AI works as an external cognitive tool that gives employees a new channel for resource input, triggering a step-by-step process from acquisition to integration to activation.

#### 2.1.3 Cognitive appraisal theory

Cognitive Appraisal Theory, which Lazarus and Folkman (1984) developed, is a well-established way to understand how people turn external events into action. Its central claim is simple: what matters is not the event itself, but how the person appraises it. This appraisal happens in two steps. Primary appraisal asks whether the event is relevant to one’s well-being. Secondary appraisal asks whether one’s coping resources are adequate (Lazarus, 1991). When people feel they have enough resources, they tend to see situations as challenges and engage in problem-solving. When they feel short on resources, they see threats and pull back into avoidance. In our model, Cognitive Appraisal Theory does most of its work at the final stage. By the time employees have gathered cognitive resources through AI use and have integrated them deeply, the appraisal process kicks in. They look at innovation situations and decide whether these are challenges worth investing in. That decision is what moves them from preparation to action.

Cognitive Appraisal Theory treats challenge and threat as two distinct evaluative responses to the same event (Lazarus and Folkman, 1984). Challenge appraisal arises when perceived resources meet or exceed situational demands; threat appraisal emerges when demands outstrip resources. In the context of generative AI use, this duality matters because AI functions primarily as an external cognitive resource. By expanding information access and solution variety, AI use increases perceived resources relative to innovation demands, thereby shifting the evaluative balance toward challenge. This does not mean threat appraisal is irrelevant. When AI use triggers identity threat, excessive cognitive load, or fear of replacement, employees may appraise innovation demands as threatening rather than challenging. Our model focuses on the challenge pathway because AI’s core affordances map onto resource gains rather than losses, but threat appraisal remains a conceptually necessary counterpart.

#### 2.1.4 The dual-theoretical integration framework

No single theory can fully explain how using generative AI leads to better innovation performance (Wang et al., 2020). COR theory is strong on the supply side. It tells us where resources come from and how they build up, but it says little about how those resources turn into actual behavior. Cognitive Appraisal Theory, on the other hand, is strong on the demand side. It explains how people assess situations and decide what to do. Yet it says little about where the resources for that assessment come from in the first place. We therefore bring the two theories together. In our framework, each theory does different work at different stages of the same chain: resource acquisition, resource integration, and resource activation. Each stage maps onto a core cognitive mechanism. Resource acquisition shows up as cognitive divergence (M1): employees use AI to expand their information search and cognitive boundaries. Resource integration shows up as cognitive elaboration (M2): employees evaluate that information critically, and then reorganize it into coherent knowledge structures. Resource activation shows up as challenge appraisal (M3): employees look at innovation situations and judge them worth pursuing, which mobilizes behavioral intentions. The theoretical division of labor across these three stages is deliberate.

Cognitive divergence and cognitive elaboration operate as cognitive processing mechanisms (Runco and Acar, 2012; Finke et al., 1992). They describe how employees acquire, expand, and reorganize information into forms useful for innovation tasks (Mumford et al., 2012), which is the resource dynamics side of COR theory. Challenge appraisal, by contrast, is an evaluative-motivational state (Moors et al., 2013; Smith and Kirby, 2001). It reflects how employees subjectively judge whether innovation demands are worth pursuing, given the cognitive resources they have built through preceding stages (Parker et al., 2010). This progression from process to state is theoretically prescribed rather than arbitrary (Ellsworth and Scherer, 2003; Gross, 2015). Cognitive elaboration converts the raw material of divergent thinking into organized knowledge structures (Nonaka, 1994), and these structures then supply the informational basis for challenge appraisal. Without this prior cognitive work, employees would have no basis for distinguishing genuine challenges from real opportunities, and their appraisals would remain superficial. The sequence therefore moves from resource construction to resource mobilization (Hobfoll, 2001), not from one cognitive stage to another.

This framework foregrounds the cognitive route because innovation is fundamentally a cognitive activity. Generative AI speaks directly to this demand through its capacity for cross-domain retrieval, solution generation, and perspective expansion. Emotional arousal and social influence may also matter, but the cognitive pathway is the most direct and the easiest to trace theoretically (Shalley et al., 2004). Other mechanisms may run alongside it, but they are not the focus here.

This framework also differs from related mechanisms in the innovation literature. Creative self-efficacy is a person-level belief, not a situation-specific appraisal (Tierney and Farmer, 2002), and absorptive capacity covers acquisition and integration but stops short of motivational activation (Cohen and Levinthal, 1990). Technology affordance perspectives describe what AI can do, not what users cognitively do with it (Leonardi, 2011).

This integrated framework sets up the hypotheses below.

### 2.2 Research hypotheses

#### 2.2.1 Generative AI use and employee innovation performance

Employee innovation performance covers the full journey of generating, promoting, and implementing novel and useful ideas at work (Scott and Bruce, 1994; Janssen, 2000). It includes idea generation, idea promotion, and idea implementation (Anderson et al., 2014). For organizations that compete in fast-moving markets, this capability is critical (Amabile, 1988; Woodman et al., 1993).

Generative AI’s main strength is its ability to produce text, code, and solutions on demand using large language models. It can pull up information, draft solutions, and compare perspectives almost instantly. These capabilities line up well with the information gathering, solution generation, and knowledge recombination that innovation demands. Empirical studies have already shown that generative AI can lift both the quantity and the quality of knowledge workers’ output (Brynjolfsson et al., 2025; Noy and Zhang, 2023).

However, having the technology is not the same as getting innovative results. To understand how AI use turns into innovation performance, two theoretical angles are useful. COR theory suggests that AI gives employees diverse information and alternative solutions that go beyond what any one person knows. It enlarges the knowledge pool they can draw on for innovative work. Halbesleben et al. (2014) found that resource investment and gain spirals predict innovative work behavior, and Bankins et al. (2024) treated AI as a new resource channel that stretches employees’ cognitive capacity. Cognitive Appraisal Theory adds another layer. When employees feel that AI bolsters their coping resources for demanding innovation tasks, they are more likely to see those tasks as challenges rather than threats. That perception fuels innovative engagement. Parker et al. (2010) found evidence that employees who feel they have adequate coping resources are more likely to engage in approach-oriented innovative behavior.

*H1*: Generative AI use is positively associated with employee innovation performance.

#### 2.2.2 The serial mediation of resource acquisition, integration, and activation

We argue that resource acquisition (cognitive divergence), resource integration (cognitive elaboration), and resource activation (challenge appraisal) form a serial mediation chain from generative AI use to employee innovation performance. Before we walk through each link, we need to address a basic question. Why treat generative AI use mainly as a resource-providing experience rather than a source of anxiety or uncertainty?

Three reasons support this framing. First, generative AI offers cognitive resources that are qualitatively different from traditional organizational resources such as supervisory support or team climate. Its core abilities include cross-domain knowledge retrieval, instant multi-solution generation, and unconventional perspective prompting. These features expand the breadth and speed of information access in ways that conventional resources simply cannot match (Brynjolfsson et al., 2025). AI acts as an external cognitive tool that builds on employees’ existing knowledge base rather than merely topping it up.

Second, COR theory’s resource investment principle (Hobfoll, 2001) says that people actively invest existing resources to acquire new ones. Using generative AI is an active investment. Employees choose to engage with these tools to strengthen their cognitive capabilities, rather than sitting back and letting technology disrupt them. This voluntary, agentic engagement sets AI use apart from involuntary stressors that mainly trigger threat appraisals.

Third, our participants are knowledge workers with at least 3 months of generative AI experience. They have moved past the initial learning curve and are therefore more likely to experience AI as empowering rather than threatening. This fits the resource gain spiral logic that early gains pave the way for later gains (Hobfoll, 2001). We do not deny that generative AI can trigger technology anxiety or identity worries when employees fear being replaced (Bankins et al., 2024). That is a valid and important threat pathway that deserves future study. Our focus here, however, is on tracing the resource empowerment pathway through which AI use promotes innovation performance.

The theoretical logic for each link is set out below.

Resource acquisition stage: Generative AI use and cognitive divergence. Cognitive divergence is the process of exploring problem solutions from multiple directions and angles (Guilford, 1967). Its hallmarks are fluency, flexibility, and originality of thought. It serves as an important cognitive foundation for innovation. Generative AI can retrieve knowledge across domains, generate multiple solutions instantly, and prompt unconventional perspectives. These capabilities let it break through the limits of any single person’s knowledge and effectively widen employees’ information search space. From a COR perspective, AI use gives employees abundant external cognitive resources. It cuts the time and effort needed to gather diverse information and starts the resource gain spiral. At the same time, the influx of varied information triggers employees to appraise new possibilities, which pushes them toward exploration and divergence.

*H2a*: Generative AI use is positively associated with cognitive divergence.

Resource integration stage: From cognitive divergence to cognitive elaboration. Cognitive elaboration is the process of critically evaluating, logically integrating, and structurally reorganizing diverse information into systematic knowledge solutions (Nonaka, 1994). The rich creative material produced during divergence supplies the raw ingredients for this deeper work. Without enough divergence, elaboration has nothing to work with. This follows the progressive logic of COR theory’s resource gain spirals. Acquiring diverse cognitive resources sets the stage for higher-order integration. When employees have abundant alternative solutions, they systematically assess the feasibility and fit of each option. They move their thinking from breadth to depth.

*H2b*: Cognitive divergence is positively associated with cognitive elaboration.

Resource activation stage: From cognitive elaboration to challenge appraisal. Challenge appraisal means employees see innovation situations as challenges worth investing in. It pushes them beyond routine work frameworks, prompts them to try new methods, and leads them to take innovation risks (Parker et al., 2010). It goes well beyond passively following instructions. It reflects employees’ willingness and ability to initiate change proactively. Cognitive elaboration fuels challenge appraisal in two ways. First, the structured knowledge solutions that deep integration produces serve as high-quality cognitive reserves. They lower the perceived risk of behavioral investment. Second, deep understanding gives employees a stronger sense of control over innovation situations. They then appraise innovation opportunities as challenges rather than threats. That appraisal activates approach-oriented innovative behavior.

*H2c*: Cognitive elaboration is positively associated with challenge appraisal.

Behavioral transformation at the resource activation stage: Challenge appraisal and innovation performance. Innovation is by nature a high-uncertainty, high-risk exploratory activity (Anderson et al., 2014). Simply having novel ideas is not enough. Without the proactivity to turn those ideas into action, innovation stalls. Challenge appraisal pushes employees to turn their internal cognitive readiness into visible innovative output. They propose new solutions to colleagues and managers, drive implementation forward, and overcome resource constraints and organizational barriers along the way (Scott and Bruce, 1994; Janssen, 2000).

*H2d*: Challenge appraisal is positively associated with employee innovation performance.

Pulling these arguments together, we see cognitive divergence, cognitive elaboration, and challenge appraisal as forming a primarily serial mediation chain in which each stage sets up the next, moving from external resource input to behavioral activation. The logic is simple: at the resource acquisition stage, COR theory explains how AI use supplies external cognitive inputs that enlarge employees’ information base. The result is a surplus of raw material. Employees now have plenty of options, but none of them fully structured yet. This surplus naturally creates pressure for the next step. Employees must evaluate and integrate these diverse inputs to produce actionable knowledge. That evaluation and integration pushes the process into the resource integration stage, where both COR theory and Cognitive Appraisal Theory work together. The outcome of integration is cognitive readiness. Employees now have structured knowledge solutions and a stronger sense of control over innovation situations. This readiness then creates the condition for the resource activation stage. Only when employees feel they have adequate cognitive resources do they appraise innovation as a challenge worth pursuing rather than a threat to be avoided. That is the appraisal-behavior logic of Cognitive Appraisal Theory (Lazarus, 1991). At the same time, we do not treat this sequential logic as a rigid pipeline. Direct and cross-stage paths are likely to coexist alongside the serial chain, reflecting the real-world complexity of human cognition. Without cognitive divergence, cognitive elaboration would have no raw material to process. Without cognitive elaboration, employees would lack the cognitive reserves needed for challenge appraisal. This progressive dependency is also built into COR theory’s resource gain spirals (Hobfoll, 2001) and into Cognitive Appraisal Theory’s assumption that appraisal quality depends on the cognitive resources one has available (Lazarus and Folkman, 1984).

*H2e*: Resource acquisition (cognitive divergence), resource integration (cognitive elaboration), and resource activation (challenge appraisal) serve as partially serial mediators between generative AI use and employee innovation performance.

#### 2.2.3 The moderating role of work stress

Work stress is the psychological tension people feel when they perceive that job demands outstrip their coping capacity (Parker and DeCotiis, 1983). In this study, we measure it as a multidimensional construct covering five pressure sources that knowledge workers commonly encounter: heavy workload and excessive tasks, role ambiguity and unclear responsibilities, conflicting job demands, strained interpersonal relationships and lack of supervisory support, and limited career development opportunities (Cooper et al., 1988; Leung et al., 2000). These pressures are not just background noise. They shape how employees direct their attention, invest their effort, and respond to new challenges at work.

We chose work stress as our focal moderator because it uniquely bridges the two theories that anchor our framework. COR theory says that under stress, people face a choice between depleting their resources and mobilizing them. When employees are swamped by workload or conflicting demands, they have stronger incentives to invest their existing resources intensively to cope. AI tools become a ready resource for doing exactly that (Hobfoll, 2001). Cognitive Appraisal Theory says that stress shapes whether people see situations as challenges that spur approach behavior or as threats that trigger avoidance (Lazarus and Folkman, 1984). When employees who feel stuck in their careers or unsupported by their supervisors discover that AI strengthens their coping capacity, they are more likely to view innovation as an opportunity rather than a burden. Few other situational variables engage both COR theory’s resource investment logic and Cognitive Appraisal Theory’s appraisal logic at the same time. That gives work stress a distinctive theoretical role in our framework.

This argument is especially relevant in the AI context. Knowledge workers often reach for AI precisely because their work demands exceed what they can handle on their own, whether through crushing workload, fuzzy role boundaries, or insufficient organizational support. In these high-pressure settings, work stress may turn AI use from casual tinkering into strategic resource deployment. Stressed employees have stronger motivation to squeeze diverse information out of AI, greater urgency to process AI-provided inputs deeply, and higher willingness to turn that knowledge into innovative action. We therefore propose that work stress interacts with AI use to amplify the efficiency of each mediation stage, as detailed below.

At the resource acquisition stage, the interaction between work stress and AI use amplifies cognitive divergence. AI use already widens employees’ information search space, but how far it widens depends on how actively employees engage with the tool. When employees are buried under heavy workload, the urgency of finding solutions pushes them to exploit AI’s retrieval capabilities more fully. They may cast a broader net rather than issuing narrow, targeted queries, although this amplification is probably weaker here than at later stages because AI’s breadth-expanding power operates fairly independently of the user’s motivation. In practical terms, the same level of AI use yields greater cognitive divergence when work stress is high, because pressure drives employees to extract more from the information resources AI makes available (Hobfoll, 1989).

*H3a*: Work stress positively moderates the relationship between generative AI use and cognitive divergence, such that higher work stress strengthens the facilitative effect of generative AI use on cognitive divergence.

At the resource integration stage, the interaction between work stress and AI use deepens cognitive elaboration. AI use supplies diverse information for processing, but whether employees process it deeply or superficially depends on their cognitive investment. When employees face role ambiguity or conflicting demands, they cannot afford to skim AI outputs lightly. Their work situations force them to resolve contradictions and clarify uncertainties. The pressure to make sense of conflicting information drives deeper evaluation and more systematic reorganization of what AI provides. That strengthens the link from AI use to cognitive elaboration.

*H3b*: Work stress positively moderates the relationship between generative AI use and cognitive elaboration, such that higher work stress strengthens the facilitative effect of generative AI use on cognitive elaboration.

**Figure 1:**
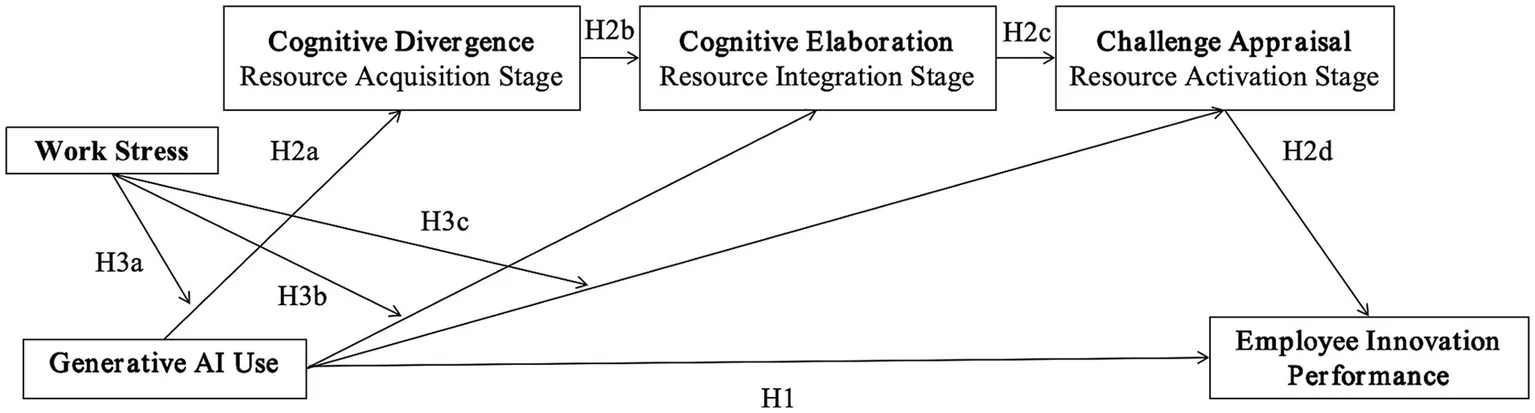
Research framework.

At the resource activation stage, the interaction between work stress and AI use intensifies challenge appraisal. AI use bolsters employees’ sense of control over innovation tasks, but whether that control translates into challenge appraisal depends on their motivation to act. When employees feel their careers have stalled or lack supervisory support, the desire for improvement primes them to seize opportunities for change. AI-enabled knowledge resources give them the capability, and stress-induced motivation gives them the drive. Together, these forces make employees more likely to see innovation as a challenge worth pursuing rather than a risk to avoid (Lazarus and Folkman, 1984) (Figure 1).

*H3c*: Work stress positively moderates the relationship between generative AI use and challenge appraisal, such that higher work stress strengthens the facilitative effect of generative AI use on challenge appraisal.

## 3 Research design and methods

### 3.1 Sample

This study targeted knowledge workers with experience using generative AI tools, collecting data through the Wenjuanxing online survey platform. This population was selected because knowledge workers’ tasks center on information processing, knowledge creation, and problem solving, all activities highly compatible with the functional characteristics of generative AI tools. The survey included a screening condition requiring respondents to have at least 3 months of generative AI tool usage experience; respondents who did not meet this criterion were excluded. Additionally, three attention-check items were embedded throughout the questionnaire (e.g., “Please select ‘strongly agree’ for this item”) to identify careless responses.

A total of 676 questionnaires were collected. Responses were excluded if they failed any attention check, had completion times below one-third of the median duration, or exhibited obvious straight-lining patterns across three or more consecutive key variables. After these exclusions, 612 valid questionnaires were retained, yielding an effective response rate of 90.5%.

Overall, respondents were predominantly knowledge workers aged 26–35 with bachelor’s degrees or above, employed in the ICT sector. The structural characteristics of this group in terms of age, education, and industry distribution demonstrate good contextual compatibility with the present study’s focus on generative AI use and employee innovation performance (Table 2).

**Table 2:** Demographic characteristics of the sample.

Variable	Category	Frequency	Percentage (%)
Gender	Male	326	53.3
	Female	286	46.7
Age	25 or below	75	12.3
	26–30	179	29.2
	31–35	187	30.6
	36–40	129	21.1
	41 or above	42	6.9
Work tenure	Less than 3 years	78	12.7
	3–5 years	149	24.3
	5–10 years	306	50.0
	Over 10 years	79	12.9
Education	High school or below	42	6.9
	Associate degree	30	4.9
	Bachelor’s degree	271	44.3
	Master’s degree	233	38.1
	Doctoral degree or above	36	5.9
Position level	Staff	409	66.8
	Junior manager	164	26.8
	Middle manager	27	4.4
	Senior manager	12	2.0
Industry	Information and communication technology	401	65.5
	Industrial and manufacturing	37	6.0
	Energy and raw materials	30	4.9
	Healthcare	31	5.1
	Finance and professional services	40	6.5
	Education and research	53	8.7
	Consumer goods and retail	4	0.7
	Construction and real estate	5	0.8
	Culture, media and creative industries	3	0.5
	Government and public services	5	0.8
	Other	3	0.5
Organization type	State-owned enterprise	85	13.9
	Private enterprise	428	69.9
	Foreign/joint venture	65	10.6
	Government/public institution	26	4.2
	Other	8	1.3
Organization size	500 or fewer	353	57.7
	500–2,000	172	28.1
	2,000–10,000	55	9.0
	Over 10,000	32	5.2
Department	Technology/R&D	545	89.1
	Product management	5	0.8
	Design/creative	5	0.8
	Marketing/branding	2	0.3
	Sales/business development	6	1.0
	Customer service/success	6	1.0
	Administration/logistics	16	2.6
	Human resources	4	0.7
	Finance/audit	2	0.3
	Legal/compliance	3	0.5
	Supply chain/procurement	1	0.2
	Operations/production	4	0.7
	Data/analytics	3	0.5
	Other	10	1.6

Percentages may not sum to exactly 100% due to rounding to one decimal place.

### 3.2 Measures

This study involved six core variables, all measured using 7-point Likert scales (1 = strongly disagree, 7 = strongly agree). All scales were adapted from established measures to fit the generative AI use context.

(1) Generative AI use. We adapted Venkatesh et al.’s (2003) use behavior scale to fit the generative AI context. The original scale comes from the Unified Theory of Acceptance and Use of Technology (UTAUT), and it is one of the most widely used instruments for measuring technology use behavior. It has shown solid predictive validity across a range of settings. Our version contains 3 items. A sample item reads: “I use generative AI tools to complete most of my work tasks.”

(2) Cognitive divergence (resource acquisition stage). We built this measure by adapting Cohen and Levinthal’s (1990) absorptive capacity scale. We kept the core dimensions (external information acquisition and transformation capacity), but we also wrote new items that tap divergent thinking. The final scale has 7 items. A sample item reads: “Using generative AI tools allows me to break out of my habitual thinking patterns.”

(3) Cognitive elaboration (resource integration stage). Adapted from van Knippenberg et al.’s (2004) information elaboration construct, comprising 7 items. The usage context was changed from wiki to generative AI tools. Sample item: “I can combine the new information provided by AI with my existing knowledge to understand problems.”

(4) Challenge appraisal (resource activation stage). Adapted from Searle and Auton’s (2015) challenge appraisal scale, comprising 9 items. The appraisal object was replaced with new challenges brought by AI use. Sample item: “I view the cognitive changes brought by AI as opportunities for growth.”

(5) Employee innovation performance. We used Janssen’s (2000) innovative work behavior scale. It contains 8 items that cover three phases: coming up with ideas, pushing those ideas forward, and putting them into practice. A sample item reads: “I am able to provide new ideas for improving the current situation.”

(6) Work stress (moderator). We adapted Cooper et al.’s (1988) Occupational Stress Indicator (OSI), drawing on Leung et al.’s (2000) standardized Chinese version. The full scale has 15 items that tap five sources of pressure: workload, role ambiguity, role conflict, interpersonal relationships, and career development. A sample item reads: “My work is complex and the workload is heavy.”

## 4 Empirical results

### 4.1 Common method bias

All variables in this study were collected through self-report questionnaires at a single time point, making common method bias a potential concern for variable relationships. To address this, both procedural and statistical remedies were employed.

For procedural controls, the questionnaire design incorporated anonymous completion, randomized item ordering, and differentiated response anchors to reduce consistency response tendencies. For statistical tests, two complementary approaches were used. First, Harman’s single-factor test showed extremely poor fit for the single-factor model (CFI = 0.505, TLI = 0.480, RMSEA = 0.110), significantly worse than the six-factor model (Δ*χ*^2^ = 5215.34, *p* < 0.001), indicating that data variance cannot be explained by a single method factor. Second, following Podsakoff et al.’s (2003) discriminant criteria, the difference between the six-factor model CFI (0.910) and single-factor model CFI (0.505) exceeded 0.200, further ruling out substantive common method bias threats. Self-reported innovation performance is inherently vulnerable to social desirability bias. However, if common method variance were the primary driver, we would expect it to inflate all paths uniformly. The observed pattern contradicts this expectation. Work stress significantly strengthened the integration and activation stages while leaving the acquisition stage unaffected. Such stage-specific effects are difficult to attribute solely to methodological artifacts.

### 4.2 Confirmatory factor analysis

Prior to confirmatory factor analysis (CFA), all scale items were examined following Hair et al.’s (2019) measurement purification guidelines. Six work stress items exhibited standardized loadings below 0.50 and were deleted based on standardized loadings and modification indices, retaining 9 items (post-deletion *α* = 0.876, CR = 0.884). Maximum likelihood estimation was then employed to conduct CFA on the six-factor measurement model, which was systematically compared with alternative models. Results are presented in Table 3.

**Table 3:** Confirmatory factor analysis: model comparison results.

Model	*χ* ^2^	df	*χ*^2^/df	CFI	TLI	RMSEA
Six-factor model (hypothesized)	1998.73	845	2.37	0.910	0.904	0.047
Five-factor A (merging M3 and innovation performance)	2949.45	850	3.47	0.836	0.826	0.064
Five-factor B (merging M2 and M3)	2934.66	850	3.45	0.838	0.827	0.063
Four-factor (merging M1 + M2 + M3)	3709.15	854	4.34	0.778	0.765	0.074
Single-factor	7214.07	860	8.39	0.505	0.480	0.110

*N* = 612. Six-factor model: generative AI use, cognitive divergence (M1), cognitive elaboration (M2), challenge appraisal (M3), work stress (9 items post-purification), innovation performance. Five-factor A: merging M3 and innovation performance. Five-factor B: merging M2 and M3. Four-factor: merging all three mediators. Single-factor: all items loaded on one factor.

As shown in Table 3, the hypothesized six-factor model demonstrated good fit (*χ*^2^ = 1998.73, df = 845, χ^2^/df = 2.37, CFI = 0.910, TLI = 0.904, RMSEA = 0.047), with all indices meeting recommended thresholds (CFI/TLI > 0.90, RMSEA < 0.06; Hu and Bentler, 1999) and clearly outperforming all alternative models, thereby supporting the discriminant validity of the six constructs.

### 4.3 Reliability and validity

As shown in Table 4, standardized factor loadings for the five core constructs ranged from 0.526 to 0.810, all significant at *p* < 0.001, indicating good convergent validity. Cronbach’s α coefficients ranged from 0.830 to 0.899, and composite reliability (CR) ranged from 0.832 to 0.900, all exceeding the recommended threshold of 0.70, demonstrating good internal consistency (Hair et al., 2019).

**Table 4:** Factor loadings, composite reliability, and average variance extracted.

Construct/item	*λ*	CR	AVE	*α*
Generative AI use		0.832	0.623	0.830
I use generative AI tools to complete most of my work tasks	0.808			
I spend most of my time collaborating with generative AI tools	0.748			
When making important work decisions, I collaborate with generative AI tools	0.810			
Cognitive divergence (M1, resource acquisition)		0.862	0.477	0.856
Using generative AI tools, I can quickly acquire needed data and information	0.702			
Using generative AI tools, I have found diverse information sources	0.751			
Using generative AI tools, I have obtained new ideas and perspectives	0.784			
Using generative AI tools has enabled me to break out of habitual thinking patterns	0.526			
I often combine seemingly unrelated information into new ideas through AI	0.729			
With generative AI tools, I can think of more ideas or solutions than usual	0.728			
By using generative AI tools, I am always stimulated to make cross-domain associations	0.571			
Cognitive elaboration (M2, resource integration)		0.899	0.559	0.898
I spend time organizing the information provided by AI	0.760			
Using generative AI tools prompts me to make more comprehensive judgments and decisions	0.751			
Using generative AI tools has enhanced my understanding of complex problems	0.767			
When using generative AI tools, I reflect on which work approaches can be optimized	0.748			
AI tools prompt me to rethink problems and analyze key points more deeply	0.769			
I can integrate new information from AI with my existing knowledge to understand problems	0.697			
With AI, I can discover previously overlooked key issues and incorporate them into my thinking	0.740			
Challenge appraisal (M3, resource activation)		0.900	0.499	0.899
I view the cognitive changes brought by AI as opportunities for growth	0.703			
AI motivates me to challenge old work methods	0.741			
I am willing to explore entirely new innovation paths with the help of AI	0.689			
The challenges brought by AI make me more motivated to unlock my potential and respond actively	0.744			
I believe I can handle the difficulties and uncertainties encountered in AI use	0.645			
Facing new problems brought by AI, I am willing to proactively try solutions	0.725			
I prefer to view the difficulties brought by AI as challenges worth investing in	0.727			
Facing the uncertainties brought by AI, I feel excited and more motivated	0.692			
When using AI, I believe I can mobilize existing resources to address new challenges	0.688			
Employee innovation performance		0.870	0.457	0.867
I can provide new ideas to improve the current situation	0.709			
I actively support innovative ideas	0.716			
I seek new work methods, skills, or tools through learning	0.666			
I have been praised by superiors for innovative ideas	0.675			
I transform innovative ideas into practical applications	0.701			
I propose original problem-solving solutions through learning	0.747			
I introduce innovative ideas using systematic methods	0.534			
I get important organizational members to pay attention to innovative thinking	0.636			

*N* = 612. λ = CFA standardized factor loading; CR = composite reliability; AVE = average variance extracted; α = Cronbach’s alpha. Work stress (9 items post-purification): *α* = 0.876, CR = 0.884, AVE = 0.465. All factor loadings significant at *p* < 0.001.

For convergent validity, generative AI use (AVE = 0.623) and cognitive elaboration (AVE = 0.559) exceeded the 0.50 threshold. Cognitive divergence (AVE = 0.477), challenge appraisal (AVE = 0.499), and innovation performance (AVE = 0.457) fell slightly below 0.50. However, combined with significant composite reliability and satisfactory loading levels, convergent validity remains acceptable. Fornell and Larcker (1981) argue that AVE thresholds should be weighed alongside composite reliability. Because every CR value in this study exceeds 0.80, the modest AVE values do not threaten construct validity. AVE is also sensitive to indicator count; scales with seven or more items often produce values marginally below 0.50 even when reliability is satisfactory (Hair et al., 2019). Moreover, discriminant validity is supported, as the square root of AVE for each construct exceeds its correlations with all other constructs (Table 5).

**Table 5:** Means, standard deviations, correlations, and square root of AVE.

Variable	*M*	SD	1	2	3	4	5	6
1. Generative AI use	4.27	1.44	(0.79)					
2. Cognitive divergence	5.16	1.20	0.36***	(0.69)				
3. Cognitive elaboration	5.40	1.22	0.41***	0.47***	(0.75)			
4. Challenge appraisal	5.41	1.15	0.42***	0.48***	0.57***	(0.71)		
5. Work stress	2.12	1.02	0.06	0.01	−0.08	−0.05	(0.68)	
6. Innovation performance	5.10	1.13	0.37***	0.43***	0.48***	0.50***	−0.08*	(0.68)

*N* = 612. Diagonal values in brackets are square roots of AVE. *p < 0.05, **p < 0.01, ***p < 0.001.

### 4.4 Descriptive statistics and correlations

As shown in Table 5, generative AI use was significantly positively correlated with all three mediators: cognitive divergence (*r* = 0.36, *p* < 0.001), cognitive elaboration (*r* = 0.41, *p* < 0.001), and challenge appraisal (*r* = 0.42, *p* < 0.001), as well as with the dependent variable innovation performance (*r* = 0.37, *p* < 0.001), providing preliminary support for theoretical expectations. For discriminant validity, the square root of AVE for each construct exceeded its correlations with all other constructs, satisfying the Fornell–Larcker criterion (Fornell and Larcker, 1981). These correlation patterns are consistent with hypothesized directions, providing preliminary support for subsequent structural equation model testing.

### 4.5 Structural equation modeling and hypothesis testing

This study employed structural equation modeling (SEM; Kline, 2016) to test the serial mediation model using maximum likelihood estimation. After controlling for gender, age, work tenure, education, and position level, the structural model demonstrated good fit: *χ*^2^ = 1443.83, df = 697, *χ*^2^/df = 2.07, CFI = 0.929, TLI = 0.924, RMSEA = 0.042. All indices met recommended thresholds (Hu and Bentler, 1999). Path coefficient results are presented in Table 6.

**Table 6:** Structural equation model path coefficients.

Path	*β* (standardized)	*b* (unstandardized)	*p*
AI use → Innovation performance	0.115*	0.093	0.015
AI use → Cognitive divergence	0.411***	0.315	<0.001
AI use → Cognitive elaboration	0.273***	0.239	<0.001
AI use → Challenge appraisal	0.183***	0.146	<0.001
Cognitive divergence → Cognitive elaboration	0.471***	0.536	<0.001
Cognitive divergence → Challenge appraisal	0.279***	0.290	<0.001
Cognitive elaboration → Challenge appraisal	0.379***	0.345	<0.001
Cognitive divergence → Innovation performance	0.183***	0.195	<0.001
Cognitive elaboration → Innovation performance	0.241***	0.225	<0.001
Challenge appraisal → Innovation performance	0.255***	0.261	<0.001

*N* = 612. β = standardized path coefficient; b = unstandardized path coefficient. Control variables: gender, age, work tenure, education, position level (all non-significant). *p < 0.05, **p < 0.01, ***p < 0.001.

Table 6 shows that all structural paths reached statistical significance. All hypothesized direct paths were supported (H1, H2a–H2d). Several cross-stage direct paths were also significant, indicating that the mediators are interrelated beyond the hypothesized serial chain. These cross-stage paths are consistent with our theoretical framework, which treats the serial chain as the primary pathway while acknowledging complementary direct and cross-stage effects. These results provide the path foundation for testing the partially serial mediation hypothesis H2e.

### 4.6 Indirect effects and serial mediation testing

To test H2e, we used Monte Carlo simulation (Preacher and Selig, 2012) with 20,000 samplings to construct 95% confidence intervals. Results are shown in Table 7.

**Table 7:** Monte Carlo test results for serial mediation indirect effects.

Indirect path	Standardized effect	Unstandardized effect	95% MC CI	Significant
AI use → Cognitive divergence → Innovation performance	0.0753	0.0614	[0.0257, 0.1016]	Yes
AI use → Cognitive elaboration → Innovation performance	0.0659	0.0536	[0.0264, 0.0861]	Yes
AI use → Challenge appraisal → Innovation performance	0.0467	0.0381	[0.0161, 0.0657]	Yes
AI use → Cognitive divergence → Cognitive elaboration → Innovation performance	0.0466	0.0380	[0.0192, 0.0609]	Yes
AI use → Cognitive divergence → Challenge appraisal → Innovation performance	0.0293	0.0239	[0.0114, 0.0404]	Yes
AI use → Cognitive elaboration → Challenge appraisal → Innovation performance	0.0265	0.0216	[0.0102, 0.0366]	Yes
AI use → Cognitive divergence → Cognitive elaboration → Challenge appraisal → Innovation performance	0.0187	0.0153	[0.0073, 0.0259]	Yes
Total indirect effect	0.3088	0.2518	[0.1720, 0.3397]	Yes

*N* = 612. Standardized effect values are products of SEM standardized path coefficients; unstandardized effect values and 95% CIs based on Monte Carlo simulation (Preacher and Selig, 2012), 20,000 parameter samplings. Significant if 95% CI excludes zero.

As shown in Table 7, all seven indirect effects were significant. The complete serial mediation path from AI use through cognitive divergence, cognitive elaboration, and challenge appraisal to innovation performance supported H2e (standardized effect = 0.0187). The total indirect effect accounted for 72.9% of the total effect, indicating a dominant mediation role; the remaining significant direct effect suggests partial mediation.

### 4.7 Alternative model comparison

To verify the superiority of the serial mediation structure, we compared the theoretical model with three alternatives. Results are shown in Table 8.

**Table 8:** Alternative structural model comparison.

Model	*χ* ^2^	df	CFI	TLI	RMSEA	Δ*χ*^2^
Model 1: Full theoretical model (partial mediation)	1238.54	517	0.929	0.923	0.048	–
Model 2: Strict sequential chain model	1392.32	522	0.914	0.908	0.052	153.78***
Model 3: Parallel mediation model	1498.87	520	0.904	0.896	0.056	260.33***
Model 4: Full mediation model (no direct effect)	1244.82	518	0.929	0.923	0.048	6.29*

*N* = 612. Model 1 = full theoretical model with all paths; Model 2 = sequential chain only; Model 3 = parallel mediation with no paths among mediators; Model 4 = full mediation with no direct effect. Δ*χ*^2^ = chi-square difference from Model 1. *p < 0.05, **p < 0.01, ***p < 0.001.

Table 8 shows that the theoretical model outperformed all alternatives. The strict sequential chain and parallel mediation models exhibited significantly worse fit, suggesting that the process is better characterized as partially serial rather than strictly sequential. The superior fit of the full model confirms that both the serial chain and complementary cross-stage paths are essential. The full mediation model showed only a slight fit reduction, consistent with the small but significant direct effect.

### 4.8 Moderation analysis

Because the full SEM is complex, we followed the standard approach and tested H3a–H3c using standardized regression interaction terms (Hayes, 2018; Aiken and West, 1991). Results are shown in Table 9.

**Table 9:** Moderating effects of work stress on AI use: mediator paths.

Dependent variable	AI use (β)	Work stress (β)	AI × Work stress (β)	*R* ^2^
Cognitive divergence	0.359***	−0.010	0.016	0.127
Cognitive elaboration	0.427***	−0.105**	0.114**	0.187
Challenge appraisal	0.438***	−0.081*	0.113**	0.193

*N* = 612. Standardized regression coefficients. *p < 0.05, **p < 0.01, ***p < 0.001.

Table 9 shows that the interaction effects differ by stage. Work stress significantly moderated the AI use–cognitive elaboration path (H3b) and the AI use–challenge appraisal path (H3c), but not the AI use–cognitive divergence path (H3a).

These results reveal a stage-dependent pattern. At the resource acquisition stage, cognitive divergence depends mainly on the AI tool’s capabilities rather than employee stress, consistent with COR theory. At the subsequent stages, work stress exerts positive moderating effects: higher stress amplifies deep information processing and challenge appraisal. This aligns with Cognitive Appraisal Theory, where stress heightens cognitive arousal and boosts mediation efficiency. We return to the non-significant H3a finding in Section 5.

**Figure 2:**
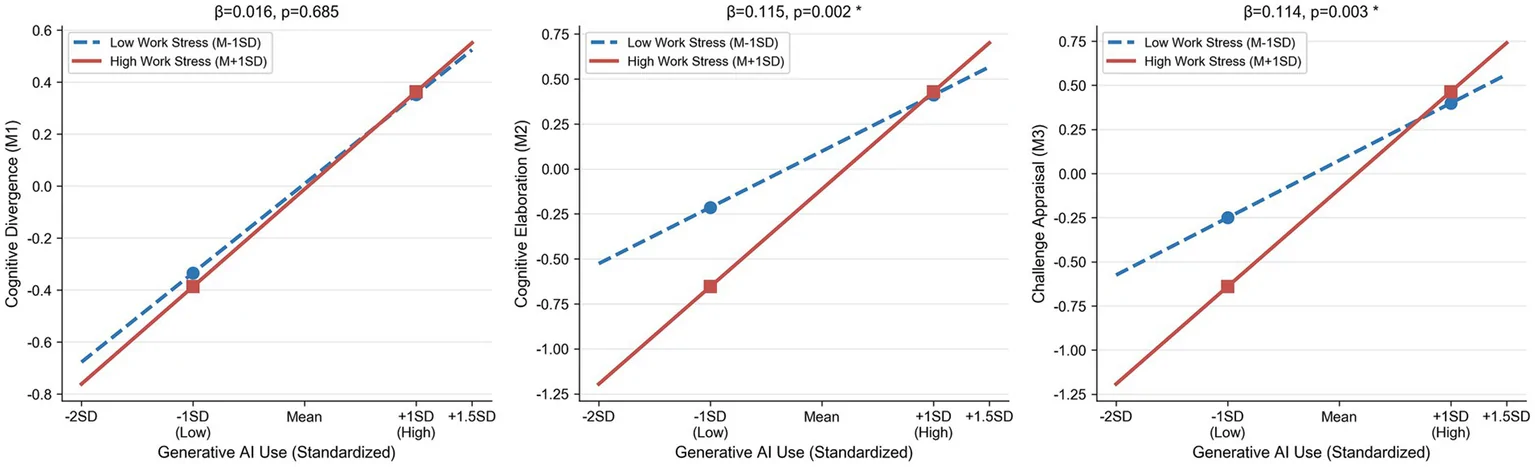
Moderating effects of work stress.

Figure 2 presents the simple slope plots for the moderating effect of work stress on the three mediator paths. At the resource acquisition stage (left panel), the slopes for low and high work stress were nearly parallel, consistent with the non-significant H3a result. At the integration stage (middle panel) and the activation stage (right panel), work stress amplified the positive relationship between generative AI use and the respective mediator, supporting H3b and H3c.

## 5 Discussion and implications

### 5.1 Research findings

We built a theoretical model that brings together Conservation of Resources theory and Cognitive Appraisal Theory. The model proposes that generative AI use is associated with innovation performance through three sequential steps: resource acquisition, resource integration, and resource activation. It also proposes that work stress moderates each step differently. Here is what we found.

First, generative AI use is positively associated with employee innovation performance. Even after we controlled for demographics, both the total effect and the direct effect were significant. H1 is therefore supported. This finding aligns with recent evidence on AI and productivity, but our context differs. Brynjolfsson et al. (2025) reported roughly a 14% efficiency gain in customer service, and Noy and Zhang (2023) showed that AI cuts task completion time and raises output quality. Yet both focus on efficiency for fairly standardized knowledge tasks, whereas innovation performance demands proactivity and tolerance for uncertainty. Our study extends the analytical lens from single laboratory tasks to multidimensional organizational innovation performance (Janssen, 2000), showing that AI use is effective for activities that call for proactive change and risk-taking.

Our positive effect differs from Dell'Acqua et al.’s (2026) jagged frontier finding, where AI helped below-average consultants but hurt top performers on complex tasks. Two factors may explain the discrepancy. First, our measure spans the entire innovation process, so aggregate effects may mask task-level variation. Second, our respondents had already adapted to AI, which may reduce over-reliance. This suggests the AI use–innovation link may be moderated by task type and user experience.

The direct effect accounted for only about one-quarter of the total effect. This means that AI use influences innovation performance mainly through internal cognitive mediation mechanisms. That pattern echoes Shalley et al.’s (2004) observation that contextual factors shape creativity primarily through individual cognitive and motivational processes. It also fits Amabile’s (1988) componential model of organizational innovation, in which external conditions must be transformed by internal individual processes before innovative outcomes appear. This gives us an empirical footing for the mediation analyses that follow.

Second, the pattern of results aligns with the three-stage partially serial mediation model. Every segment of the chain reached statistical significance (H2a–H2d all received support). Monte Carlo confidence intervals for all indirect effect paths excluded zero, and the complete partially serial mediation path was significant (H2e supported). The total indirect effect accounts for over 70% of the total effect, suggesting that the mediation mechanism carries most of the explanatory weight.

This finding advances the literature beyond earlier one- or two-mediator models. In innovation research, single mediators have long been the norm; Tierney and Farmer (2002), for example, identified creative self-efficacy as the core mediator. Zhang and Bartol (2010) built a longer chain, yet it centers on motivational activation rather than the cognitive transformation of external resources. Neither traces the full path from external resource input to behavioral output through multi-stage cognitive change. Classic creativity research has also focused mainly on direct effects (Oldham and Cummings, 1996; Zhou and George, 2001). Our three-stage model fills this gap.

Our model also speaks to Perry-Smith and Mannucci's (2017) idea journey framework, which conceptualizes innovation as moving through generation, elaboration, championing, and implementation. We offer micro-level empirical support from a cognitive angle: cognitive divergence maps onto generation, cognitive elaboration onto elaboration, and challenge appraisal onto the behavioral activation carrying elaboration into implementation. The dominant indirect effect further confirms that AI-enabled innovation is a gradual multi-stage transformation rather than a sudden flash.

From a COR theory perspective, the serial chain supports Hobfoll’s (2001) resource gain spiral proposition. Earlier empirical work has tested this idea mainly in stress coping and burnout contexts (Hobfoll et al., 2018). We extend it to the AI-assisted innovation context. Our results show that the cognitive divergence triggered by AI can be progressively amplified through cognitive elaboration and challenge appraisal, ultimately translating into innovation performance. That broadens the scope of resource gain spirals and adds new evidence on how resources behave in technology-empowered workplaces.

Third, work stress moderates the chain in a stage-dependent gradient. It is silent at the resource acquisition stage but significantly positive at the integration and activation stages. Compared with LePine et al.’s (2005) challenge-hindrance framework, this pattern extends their logic by showing that the same stressor’s effect depends not only on its type but on which cognitive stage it operates in. It also reframes Baer and Oldham’s (2006) curvilinear pressure-creativity relationship as a possible aggregate of stage-specific linear effects rather than a uniform nonlinear effect.

At the resource acquisition stage, mediation efficiency hinges on tool capabilities rather than stress perceptions, so individual differences have limited moderating room. At the integration and activation stages, stress heightens cognitive arousal and deepens processing. These positive effects suggest that employees with AI-enabled resources appraised heightened pressure as a challenge, which aligns with the premise that resource adequacy shapes evaluative valence. Yet this pattern is bounded. Without sufficient AI-derived resources, or when AI use triggers overload or identity threat, the same pressure would likely tilt appraisal toward threat. Our findings therefore illuminate one set of conditions that favor challenge over threat, though they do not preclude the complementary threat pathway.

Null moderation at acquisition and significant moderation at integration and activation point to a tool-driven versus person-driven distinction. At the resource acquisition stage, generative AI operates as an external capability amplifier. Its output generation depends on algorithmic capacity rather than the user’s transient motivational state, so employees under high stress still obtain cognitive divergence. Yet moving from acquisition to integration and then to activation demands intrinsic resources such as selective attention, working memory, and creative confidence, which stress specifically depletes. In COR terms, technological resources buffer against loss at the input stage, but human resources remain vulnerable at transformation and deployment. Stress therefore creates a bottleneck not where resources enter the system, but where they must be converted and acted upon.

### 5.2 Theoretical contributions

Our study makes theoretical contributions in three areas for generative AI and employee innovation research.

First, the partially serial framework addresses the transformation problem in human-AI collaboration research: how AI-enabled cognitive resources are converted into innovative behavior. Existing accounts each capture part of this conversion but stop short of the full chain. Technology affordance perspectives describe what AI tools enable users to do (Leonardi, 2011), yet they do not specify the cognitive processes through which users convert these affordances into innovative output. Absorptive capacity models illuminate how employees recognize and assimilate external knowledge (Cohen and Levinthal, 1990), but they lack a motivational bridge to behavioral activation. Creative self-efficacy frameworks center on person-level beliefs about creative capability (Tierney and Farmer, 2002), yet they do not address how AI interaction reshapes the cognitive pathway in the moment. Zhang and Bartol (2010) built a longer motivational chain, yet it does not trace the cognitive transformation of external resources. None of these approaches specifies the intermediate stages through which raw AI outputs are progressively transformed into innovative behavior. By introducing three progressive mediators and verifying seven indirect effect paths, we provide a systematic account of this conversion.

Second, our dual-theoretical integration framework. Past attempts to combine COR theory and Cognitive Appraisal Theory have mostly stacked one theory on top of the other without real integration. We instead embed both within different stages of the same mediation chain, allowing each theory to carry different explanatory loads at different points. The stage-dependent pattern of work stress moderation offers indirect support for this division of labor. This approach moves beyond a simple side-by-side application at the same explanatory level. It arranges the two theories sequentially across different levels of psychological analysis, from cognitive processing to evaluative state, and in doing so traces the complete arc from resource input to behavioral output. It also moves beyond isolating challenge appraisal. By specifying the resource conditions that favor challenge over threat, our framework positions threat appraisal as the theoretically prescribed alternative when those conditions break down.

Third, we identify the stage-dependent differential moderation of work stress within the AI-enabled innovation chain. Past research has concentrated on the overall directional effect of stress on performance (LePine et al., 2005) or on curvilinear relationships (Baer and Oldham, 2006). It has paid little attention to how the same stress variable behaves differently at different stages. We find that work stress is constrained by the dominant theoretical logic at each stage. It is not significant at the resource acquisition stage, which is driven by external tools, but it is significantly positive at subsequent stages, which are driven by subjective cognitive appraisal. This contributes in two ways. It extends LePine et al.’s (2005) framework by adding mediation stage characteristics as a complementary analytical dimension alongside stressor type. And it gives a micro-level explanation for Baer and Oldham’s (2006) overall curvilinear pattern: the aggregate nonlinear effect may simply be the sum of different stage-specific effects stacked across stages. This tool-driven versus person-driven distinction offers a boundary condition for predicting when individual-difference variables will moderate human-AI relationships.

### 5.3 Practical implications

Our findings carry practical implications for organizations that want to optimize their generative AI deployment and foster employee innovation. These recommendations rest on a sample of predominantly young, highly educated knowledge workers in the ICT sector who use generative AI voluntarily, and their fit to other populations should be tested before broad application.

First, shift from tool promotion to resource empowerment. Because AI use links to innovation performance mainly through internal resource mediation mechanisms, simply handing employees AI tools appears insufficient in this context. Managers should help employees use AI for multi-dimensional information exploration (strengthening resource acquisition), learn methods for turning AI outputs into structured solutions (promoting resource integration), and build the awareness and skill needed to turn knowledge into innovative action (stimulating resource activation). Systematic training and communities of practice can support all three stages.

Second, manage stress differently at different cognitive stages. The stage-dependent moderating effects of work stress tell us that moderate stress was associated with deeper cognitive investment in this sample and innovative behavior transformation at the resource integration and activation stages, even though excessive stress may still drain resources (Hobfoll et al., 2018). Managers could foster a moderately challenging work climate. They can set meaningful innovation goals that spark cognitive arousal, but they should also provide enough organizational support to keep stress from becoming a hindrance.

Third, build innovation environments that support knowledge sharing and cross-domain collaboration. Our three-stage model shows that progression from resource acquisition to resource activation is sequential. A breakdown at any stage can weaken the final output. Organizations might consider supporting each stage through institutional safeguards and collaborative infrastructure. Illustrative directions include AI-assisted knowledge management platforms or cross-departmental AI experience exchanges, though their causal impact awaits experimental verification.

### 5.4 Limitations and future directions

This study has the following limitations, which also point to directions for future research.

First, the cross-sectional design makes it difficult to establish strict causal relationships. Although theoretical reasoning, alternative model comparison, and multiple statistical tests enhance the plausibility of causal inferences, future research could adopt multi-wave longitudinal designs or quasi-experimental designs to more rigorously verify the temporal mediation logic of the three stages.

Second, all variables were collected through employee self-report. Although statistical tests ruled out substantive common method bias threats (Podsakoff et al., 2003), the convergence of mono-method data with modest AVE values warrants cautious interpretation of effect magnitudes. Future research could introduce multi-source data, such as supervisor ratings of innovation performance and system logs recording AI use patterns, to enhance measurement objectivity.

Third, the sample boundaries limit generalizability. Respondents were predominantly aged 26–35, held bachelor’s degrees or above, worked in the ICT sector, and had chosen to use generative AI for at least 3 months. This profile matches the population most likely to encounter GenAI in their work today, making the sample appropriate for testing early adoption dynamics. However, it limits generalizability to employees in traditional industries, older workers, those with lower digital literacy, or settings where GenAI exposure is mandatory rather than voluntary. Future research could test these relationships in manufacturing, public sector, and cross-cultural settings.

Fourth, we measured challenge appraisal but not threat appraisal. Our theoretical reasoning and sample context support the dominance of the challenge pathway in this study, yet we cannot rule out the possibility that threat appraisal co-occurs or suppresses innovation under different conditions, such as mandatory AI adoption or high job insecurity. Future research would benefit from including both valences to test how resource conditions determine their relative strength.

Fifth, work stress was analyzed as a unidimensional construct without distinguishing between challenge and hindrance stressors. Future research could decompose work stress into two dimensions (LePine et al., 2005) to examine whether different stressor types exhibit more refined moderating patterns across the three mediation stages.

### 5.5 Conclusion

Generative AI is now deeply woven into knowledge work, and our study addresses the central question of how this technology relates to employee innovation performance through cognitive processing. We constructed and tested a three-stage serial mediation model of resource acquisition, resource integration, and resource activation. Three principal findings emerge. Generative AI use is associated with innovation performance through the progressive pathway of cognitive divergence, cognitive elaboration, and challenge appraisal. Indirect effects dominate, which suggests that AI-enabled innovation is fundamentally a multi-stage resource transformation process. And work stress moderates the chain in a stage-dependent way, revealing that stress effects are tied to the cognitive stage at which they operate. These findings push forward the application of resource gain spirals in COR theory, propose a workable dual-theoretical explanatory paradigm, and give organizations evidence-based guidance for optimizing AI deployment and managing stress in a differentiated way. As generative AI technology keeps evolving, future research could extend these findings through longitudinal causal tracking, cross-cultural validation, and finer-grained stressor type differentiation.

## Data Availability

The original contributions presented in the study are included in the article/supplementary material, further inquiries can be directed to the corresponding author.
